# Assessment of Waveform Similarity in Clinical Gait Data: The Linear Fit Method

**DOI:** 10.1155/2014/214156

**Published:** 2014-07-13

**Authors:** M. Iosa, A. Cereatti, A. Merlo, I. Campanini, S. Paolucci, A. Cappozzo

**Affiliations:** ^1^Clinical Laboratory of Experimental Neurorehabilitation, IRCCS Fondazione Santa Lucia, Via Ardeatina 306, 00179 Rome, Italy; ^2^Information Engineering Unit, POLCOMING Department, University of Sassari, Viale Mancini 5, 07100 Sassari, Italy; ^3^Motion Analysis Laboratory, AUSL of Reggio Emilia, Rehabilitation Department, Via Circondaria 26, Reggio Emilia, 42015 Correggio, Italy; ^4^Interuniversity Centre of Bioengineering of the Human Neuromusculoskeletal System, Department of Movement, Human and Health Sciences, University of Rome “Foro Italico”, Piazza Lauro de Bosis 6, 00194 Rome, Italy

## Abstract

The assessment of waveform similarity is a crucial issue in gait analysis for the comparison of kinematic or kinetic patterns with reference data. A typical scenario is in fact the comparison of a patient's gait pattern with a relevant physiological pattern. This study aims to propose and validate a simple method for the assessment of waveform similarity in terms of shape, amplitude, and offset. The method relies on the interpretation of these three parameters, obtained through a linear fit applied to the two data sets under comparison plotted one against the other after time normalization. The validity of this linear fit method was tested in terms of appropriateness (comparing real gait data of 34 patients with cerebrovascular accident with those of 15 healthy subjects), reliability, sensitivity, and specificity (applying a cluster analysis on the real data). Results showed for this method good appropriateness, 94.1% of sensitivity, 93.3% of specificity, and good reliability. The LFM resulted in a simple method suitable for analysing the waveform similarity in clinical gait analysis.

## 1. Introduction

Instrumented gait analysis (GA) allows for gathering quantitative information about joint kinematics and kinetics of the musculoskeletal system during gait. A critical issue in the field of human movement analysis refers to the data comparison and the assessment of the deviation of the gait pattern under analysis from reference data through a few meaningful indices [1]. The need to assess the similarity between two curves of gait data is encountered in a large variety of different scenarios, for example when two datasets are obtained using different instrumentations [2–4], using different protocols [5–7], before and after treatment [8, 9], or especially for comparing pathological gait data related to a patient (or a group of patients) to physiological gait data related to healthy subjects [10–12].

In general, the comparison between pairs of kinematic or kinetic curves is performed by computing the Pearson correlation coefficient (*R*), which allows for quantifying the strength of a linear relationship between the two curves (i.e., their shape similarity, independently from their amplitude or their mean difference) [2, 13], the root mean square error (RMSE) which provides a positive global index [2], or the coefficient of multiple correlation (CMC) which is helpful when the reliability of a group of curves is under analysis [14, 15].

Another simple approach consists in computing the difference between parameters assumed to be representative of the entire curve, such as the range of motion (ROM), or computing the mean difference between the mean values of the curve under analysis (*P*_*a*_) in respect of the mean value of the reference curve (*P*_ref_). Some indices were proposed to compare the ROM of the two curves in order to quantify the differences in terms of pattern amplitude, such as the ratio index (RI_ROM_ = ROM_Pa_/ROM_Pref_) [16] or the symmetry angle [17]. Conversely, the mean difference ($\text{MD}=\overset{-}{P_{a}}\;-\;\overset{-}{P_{\text{ref}}}$) is used to assess a vertical shift (offset) between the curve under analysis and the curve used as reference [2].

More complex methods have also been suggested, including extended indices [18], Fourier analysis [19], principal component analysis [20], eigenvectors [21], fractal methods [22], neural networks [23], pattern recognition techniques [24], or ninth order polynomial fitting [25].

Another approach commonly adopted in gait analysis consists in calculating the differences between the curves in correspondence of specific gait events (e.g., at foot contact or at foot off). However, for each curve several points can be extracted and their selection could be arbitrary, and it implies a loss of information about the entire pattern and hence it can be critical.

Finally, it is possible to combine the above described approaches, such as in the study of Crenshaw and Richards [21], who suggested to compute five parameters three of which based on eigenvectors computation, and the other two being MD and RI_ROM_.

There is hence a large amount of research in the field, but the multiplicity of proposed methods suggests that a simple approach to perform a global comparison between gait curves through a few parameters with specific physiological meaning is still lacking. The aim of this study is to present and validate a simple method based on linear fitting applied to two datasets plotted one versus the other (linear fit method, LFM). This method was preliminary suggested by our group [26] and then, despite not being validated yet, applied for comparing gait data obtained in different laboratories [27]. Presented here is the validation of LFM, carried out in terms of appropriateness, sensitivity, specificity, and reliability.

## 2. Material and Methods

### 2.1. Analytical Description of the Linear Fit Method

**Figure 1:**
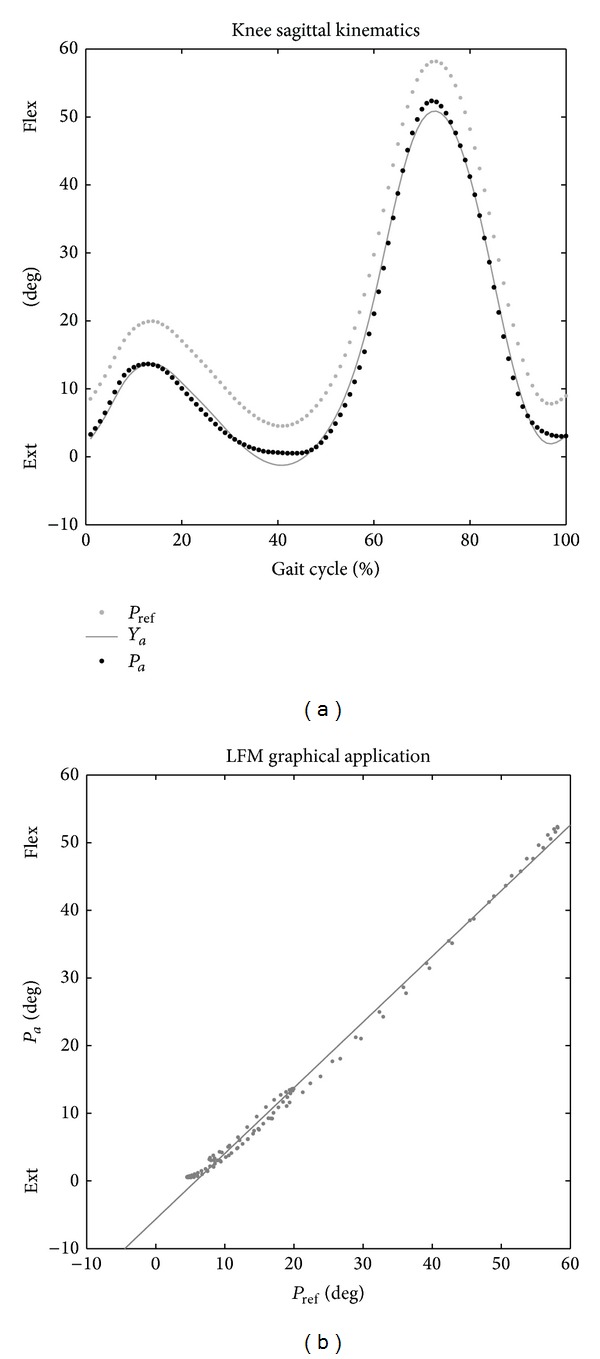
Two exemplificative knee sagittal kinematic datasets were compared in order to graphically illustrate the LFM. The circles represent the 100 values obtained for the two knee kinematics when time-normalized and reported in terms of gait cycle. On the left are the points for the investigated dataset *P*_*a*_ (black dots) and for the reference dataset *P*_ref_ (grey dots). The grey line represents the reconstructed curve *Y*_*a*_ obtained by the parameters of the linear fit applied to the values of *P*_*a*_ when plotted versus *P*_ref_ (right plot).

Let *P*_*a*_ be the kinematic (or kinetic) dataset under investigation that should be compared with *P*_ref_, that is, the reference dataset (as shown in the left plot of Figure 1). As usual in GA, the datasets are time-normalized between two selected events, such as two consecutive foot-strikes of the same limb which define the gait cycle [28]. Since *P*_*a*_ and *P*_ref_ are normalized in respect of gait cycle, they result in two arrays of real numbers of the same length. It is hence possible to plot *P*_*a*_ against *P*_ref_, to define a set of points in a Cartesian coordinate system ((*X*_*i*_; *Y*_*i*_)  with  *i* = 1 … *N*) where *P*_ref_ values correspond to *x*-values and *P*_*a*_ to *y*-values (Figure 1). The present method is based on applying a linear fit to this set of points. This fitting minimizes, in a least squares sense, the sum of the square vertical distances between the points and the fitting line (regression line, right plot of Figure 1):

(1) Ya=a1·Pref+a0,

where *Y*_*a*_ represents the linear function which approximates *P*_*a*_ values by means of a linear transformation of values of *P*_ref_; *a*_1_ is the angular coefficient; and *a*_0_ is the intercept of the fitting line. The goodness of the fit can be easily assessed by the coefficient of determination *R* ^2^ which coincides with, for the properties of linear fit, the square of the Pearson's correlation coefficient *R*.

The LFM relies on the interpretation of the values of *R* ^2^, *a*_0_, and *a*_1_ for assessing curve similarity between *P*_*a*_ and *P*_ref_.

The formulas for computing the above three parameters are

(2) a1=∑i=1N(Pref(i)−Pref¯)·(Pa(i)−Pa¯)∑i=1N(Pref(i)−Pref¯)2,a0=Pa¯−a1·Pref¯,R2=∑i=1N(a0+a1·Pref(i)−Pa¯)∑i=1N(Pa(i)−Pa¯)2,

with *N* the length of datasets (corresponding to the 100% of gait cycle) and overline used for indicating the mean value of a dataset.

The meaning of LFM parameters is described below.

- *a*_1_ measures the mean variation of *P*_*a*_ for every one-unit change in *P*_ref_. It hence represents the amplitude scaling factor, that is, the factor for which *P*_ref_ should be multiplied to match *Y*_*a*_ except for a scalar addition.
- *a*_0_ predicts this scalar addition (shift), that is, the value of *P*_*a*_ when *P*_ref_ is equal to 0.
- *R* ^2^ measures the strength of the linear relationship between *P*_*a*_ and *P*_ref_, that is, the percentage of variance in *P*_*a*_ that can be matched by the variance in *P*_ref_.

It should be noted that if *P*_*a*_ = *P*_ref_ then the values of LFM parameters are *a*_1_ = 1, *a*_0_ = 0, *R* ^2^ = 1. Further, the LFM has the advantage that, for its intrinsic linearity, the mean *a*_1_- and *a*_0_-values obtained from *n* comparisons of *n* different *P*_*a*_-data sets with their mean pattern are equal to the ideal values: *a*_1_ = 1 and *a*_0_ = 0. This is the case when *n* curves of healthy subjects are compared with a reference pattern obtained as their mean.

### 2.2. LFM Validation

To describe the application and the advantages of LFM in comparison with other parameters commonly used in GA, we first analyzed synthetic datasets generated from a real reference pattern (a physiological knee sagittal kinematics) in which mathematical transformations were applied in order to simulate specific gait pattern alterations. Synthetic data were used to allow for perfectly knowing the mathematical difference between *P*_*a*_ and *P*_ref_.

LFM was then validated using real data in terms of (a) appropriateness (does it provide different results for patients when compared to healthy subjects?), (b) sensitivity (does it detect a specific difference in the curves?), (c) specificity (is it able to detect as pathological only actually pathological patterns?), and (d) reliability (can the measures be repeated accurately?).

In particular, to test the capacity of LFM to detect a difference when it is present, we have applied it on five different synthetic arrays of data (*Y*_*i*_,* i* from 1 to 4) obtained by altering the mean knee sagittal kinematics (*P*_*a*_) obtained from 15 healthy subjects acquired by means of a stereophotogrammetric system during level walking. The mathematical reshape of these reference data allowed for examining the variation of LFM parameters when one (or more) specific feature of the curve was selectively altered in order to simulate a specific knee impairment (*Y*_1_: hyperextended knee, *Y*_2_: knee with reduced mobility, *Y*_3_: stiff knee, *Y*_4_: hyperflexed knee). For these curves, values of the three parameters obtained using LFM were compared with the values of three parameters commonly used in literature: RI_ROM_, MD, and RMSE.

To assess the LFM appropriateness, the data relative to the sagittal kinematics of hip, knee and ankle of 15 healthy subjects were compared with those of 34 patients affected by cerebrovascular accident (CVA). The values of LFM parameters obtained for the two groups were hence compared by means of unpaired 2-tailed *t*-tests. For this and all the other statistical tests applied in the present study the threshold for statistical significance was set at 0.05.

Relevant mean, standard deviation, and 95% confidence interval (IC_95%_) for each set of data were also computed.

LFM sensitivity and specificity were assessed performing a Wilks' lambda discriminant analysis computed on the above described real data. This analysis was performed to assess the capacity of LFM to cluster the subjects into two groups: healthy group and patient group.

The reliability of LFM was evaluated by computing the intraclass correlation coefficient (ICC (2, 1)) for hip, knee, and ankle sagittal kinematics.

Gait datasets were acquired using a 9-camera motion capture system (Smart-D system, BTS Bioengineering, Milan, Italy) to reconstruct the 3D position of 21 retroreflective spherical markers located on the subjects skin according to the conventional method [29], during level walking in barefoot conditions at self-selected speed. Datasets were related to three trials per side for each one of fifteen healthy subjects and six trials of affected side for each one of the 34 patients with CVA.

## 3. Results

### 3.1. Comparison of LFM Parameters to Other Parameters on Synthetic Data

**Figure 2:**
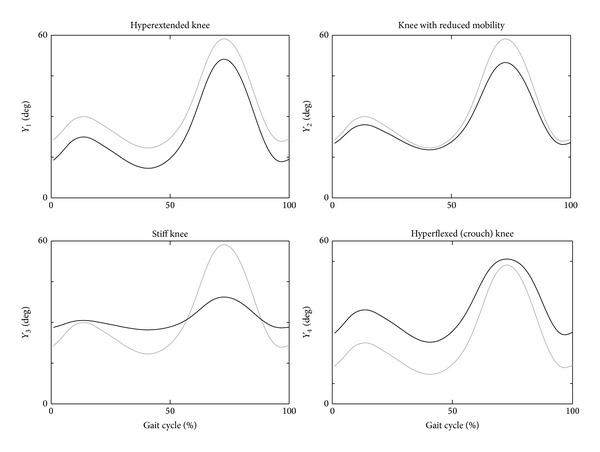
Four synthetic reshapes (black lines) mathematically obtained by a knee reference pattern (grey line), simulating four different impairments.

Figure 2 shows four synthetic reproductions of knee impairment, and Table 2 reported the relevant mathematical equations applied to obtain these data, together with the values of computed parameters. The values obtained for RI_ROM_, MD, and RMSE were misleading and less meaningful in respect of the values of LFM parameters. In detail, the analysis performed on the synthetic datasets *Y*_2_ and *Y*_3_ highlighted that when the offset between curves is evaluated using MD, its value is influenced by the amplitude differences (Table 1), whereas the LFM evaluated the shift independent of amplitude differences, by *a*_0_ and *a*_1_, respectively. The RI_ROM_ and *a*_1_ values were very similar for the analysed synthetic data, despite *a*_1_ values were not dependent by the artefacts affecting the values of RI_ROM_. The RMSE values ranged between 10° and 15° for three out of four investigated datasets (*Y*_1_, *Y*_3_, *Y*_4_), despite that the differences were due to an offset in *Y*_1_, to a combination of offset and amplitude differences in *Y*_3_ or to shape dissimilarity in *Y*_4_. In this last case, the mathematical transformation was not linear, but the value of *R* ^2^ remained high, and the waveform differences were better quantified by parameters of LFM than by RI_ROM_, MD, and RMSE.

### 3.2. Appropriateness

LFM has been applied to analyze the sagittal kinematics of healthy subjects (walking at 68 ± 10% of their stature/s) and of patients with CVA (walking at 42 ± 15%, *P* < 0.001). Mean, standard deviation, and confidence interval of the sagittal hip, knee, and ankle kinematics of the healthy subjects group were reported in Table 2. For healthy subjects, the mean *a*_1_ and *a*_0_ resulted to be equal to ideal values 1 and 0, respectively, whereas the mean *R* ^2^ was just close to its ideal value for hip (0.99), knee (0.97), and ankle (0.89) joint (in mean 0.95). The statistical analysis reported in Table 2 showed that the values of the LFM parameters resulted to be statistically different between patients and healthy subjects. In detail, for patients *R* ^2^ resulted to be significantly lower at all the three joint levels, the movement amplitude resulted to be significantly reduced for all the three joints (*a*_1_ ≤ 0.70), and the hip resulted to be hyperflexed (*a*_0_ = 6.7°).

### 3.3. Sensitivity and Specificity

The correctness of classification in physiological versus pathological patterns obtained by LFM prediction was 79.4%, 76.5%, and 94.1%, respectively, for hip, knee, and ankle data (in mean 83.3%). It means that only 2 patients out of 34 (5.9%) were classified as without any impairment. For healthy subjects, among all the three joints, only one false positive was found: a knee pattern of one healthy subject was classified as pathological. Hence, the specificity resulted to be of 93.3% (true negative rate: 14/15) and sensitivity resulted to be of 94.1% (true positive rate: 32/34).

### 3.4. Reliability

The reliability of parameters computed for healthy subjects and patients was assessed by means of the intraclass correlation coefficient computation. The mean ICC values for patients are shown in Table 3 (mean value: 0.91 ± 0.07). The reliability was lower for healthy subjects (mean value: 0.69 ± 0.21). A similar trend was observed for the walking speed; the value of ICC was higher in patients (0.91) than in healthy subjects (0.88).

## 4. Discussion

The aim of our study was to present and validate a linear fit method for assessing the similarity between curves relative to gait datasets. This assessment is usually the basis of GA, both for clinical and research purposes.

The results obtained in this study on synthetic data showed that the values with conventionally used parameters, such as MD, RI_ROM_, and RMSE, can be misleading. Conversely, *a*_0_ and *a*_1_, two of the LFM parameters, can be used as representative of offset and amplitude difference, respectively, without the following problems affecting the values of MD and RI_ROM_. For example MD, which is generally adopted to assess the presence of a vertical shift, can be potentially affected by changes in amplitude (as evident for *Y*_2_ and *Y*_3_). The problem of vertical shift is particularly important, for example, when tests were repeated and GA-markers need to be replaced (potentially introducing an offset): this shift is the most important factor in reducing the reliability of repeated measures and it should be properly assessed [27, 30]. On the other hand, the use of ROM (and hence RI_ROM_ and other related indices) can have some disadvantages: (1) it only compares the differences between the maximum and the minimum of the curves, independently from the data distribution; (2) the mean ROM of *n* curves can be very different from the ROM of the relevant mean curve, and it can affect the assessment of amplitude similarity. The recovery of a functional ROM is an important outcome measure in rehabilitation. Differently from ROM, *a*_1_ takes into account the amplitude of the gait pattern in respect of the physiological pattern along the entire gait cycle. The RMSE has the problem that its values were similar over the different conditions (such as synthetic data *Y*_1_, *Y*_3_, *Y*_4_) and hence its physiological meaning is difficult to be argued. More clear is the meaning of Pearson correlation coefficient *R* (despite being improperly used in many studies as an indicator of agreement of two datasets) [31, 32]. However the same information obtained with *R* can be obtained in LFM by the *R* ^2^ value, which provides a measure of the shape similarity of two curves with a clear mathematical meaning (the percentage of variance of the dataset under analysis explained by a linear transformation of the reference dataset) and a clear physiological meaning (the pattern similarity despite possible amplitude differences or presence of a shift). *R* ^2^ resulted in an index for summarising the waveform similarity, and it can potentially quantify the efficacy of a treatment in relationship to functional and structural recovery as indicated by ICF [33].

The LFM have some advantages clearly reported and that could be summarised as follows: (1) LFM takes into account all data point distributions (resulting in less dependence on single peak-values than ROM and other similar parameters); (2) LFM is simple to be applied; (3) this simplicity implies a clear meaning of its few (three) parameters; (4) its linearity implies that mean parameter values of *a*_0_ and *a*_1_ are equivalent to parameter values of the mean curve; (5) this linearity also allows for using powerful parametric statistics; (6) because *a*_1_, *a*_0_, and *R* ^2^ were computed at the same time, they independently assess curve differences in terms of amplitude, offset, and similarity, respectively.

We have validated the LFM on kinematic data, but this validation is clearly extensible to kinetic or even electromyographic data because they are all usually time normalized and reported in terms of gait cycle. Our validation showed LFM is appropriate to discriminate between patients and healthy subjects, showing a good sensitivity in identifying pathological gaits and a good specificity (only one false classification out of 15 in the pathological cluster definition). The reliability of LFM parameters was found high, especially for patients and even for the ankle which was characterized by low *R* ^2^ values. The lower ICC found for healthy subjects is not surprising. In fact, in healthy populations the intersubjects variability is similar to intrasubject variability, resulting in a low ratio between intra- and intersubject variability, as it was already highlighted [10].

In this study, we compared datasets obtained using a stereophotogrammetric system, according to the conventional gait analysis. In the last decade, alternative approaches have been developed based on the use of wearable sensors (including accelerometers, gyroscopes, and magnetic units) [2, 34–37]. In this study, we did not test datasets obtained using these devices; however, it is conceivably that LFM could be useful for comparing the results of these new approaches with those obtained with the conventional gold standard (i.e., the stereophotogrammetric system).

The proposed LFM have some limitations which should be considered and which can bound its fields of application: (1) the values of *a*_0_ and *a*_1_ lose meaning when the linear relationship between *P*_*a*_ and *P*_ref_ is poor (low value of *R* ^2^); (2) there is the need for identifying one of the two dataset as the reference one (*P*_ref_); (3) a bias can be introduced by the presence of a phase shift. First, LFM relies on the hypothesis that two gait patterns related to a specific joint are usually characterized by a similar waveform, given the intrinsic biomechanical constraints of the musculoskeletal system. In this respect, it is necessary to define when the hypothesis of linear relationship decays. Despite *R* ^2^ being high even for nonlinear transformations, such as in synthetic data *Y*_4_ or for hip and knee joints in real data, it should be taken into account that for *R* ^2^ < 0.50 (i.e., less than 50% of *P*_*a*_ variance matched by *P*_ref_ variance, corresponding to a *R* < 0.70) the relationship between *P*_*a*_ and *P*_ref_ can be only partially described by means of a linear transformation and hence the values of *a*_1_ and *a*_0_ should be carefully handled. Second, the results of LFM depend on which dataset, between *P*_*a*_ and *P*_ref_, is chosen as reference. This problem is common also to other methods. For LFM, the optimization of the fitting is determined by minimizing vertical distances between points obtained from plotting *P*_*a*_ versus *P*_ref_ and fitting a line (see Figure 1). This characteristic may represent a potential limitation of the method, although the existence of a clearly defined *P*_ref_ is a common circumstance in most clinical applications (e.g., in comparing the pathological gait patterns to normative data). However, when the reference data set chosen is questionable, such as symmetry assessment in healthy subjects [17], LFM could still be used, but for applying a fit minimizing the orthogonal distances between points and fitting a line. Finally, we would highlight that when a phase shift between *P*_*a*_ and *P*_ref_ is present (i.e., a shift along the horizontal gait cycle axis), this results in a reduction of shape similarity. Since gait curves are usually time normalized and expressed as a percent of the gait cycle this aspect should not be critical in most of the cases, but if a phase shift is present we suggest to previously perform a cross-correlation for quantifying the horizontal shift and then apply the LFM to the realigned curves.

In conclusion, the strengths and attractiveness of the proposed linear fit method are its easy mathematical implementation, the use of a few parameters, their straightforward physical interpretation, and their evident clinical meaning.

## Figures and Tables

**Table 1:** Parameter values for the curves shown in Figure 2.

Pattern	RI_ROM_	MD	RMSE	Linear fit method		
				*a*_1_	*a*_0_	*R* ^2^
*Y*_1_ = *P*_*a*_ − 10°	1	−10°	10°	1	−10°	1
*Y*_2_ = 0.8∗*P*_*a*_	0.8	−4°	6°	0.8	0	1
*Y*_3_ = 0.3∗*P*_*a*_ + 15°	0.3	0°	12°	0.3	15°	1
*Y*_4_ = −0.007∗*P*_*a*_^2^ + 1.2∗*P*_*a*_ + 15°	0.76	14°	15°	0.77	19°	0.98

**Table 2:** Mean ± standard deviation (SD) of the values of LFM parameters for normative data obtained by healthy subjects and relevant values for patients with CVA. For healthy subjects the 95% interval of confidence (IC_95%_) is reported, whereas for patients, the *P* value of comparison with healthy subjects' values is reported. *R* ^2^ and *a*_1_ are adimensional coefficients, whereas *a*_0_ is measured in degrees.

Mean ± SD (IC_95%_)	Hip	Knee	Ankle
Mean ± SD (IC_95%_) of healthy subjects			
*R* ^2^	0.99 ± 0.01	0.97 ± 0.02	0.89 ± 0.06
	(0.98; 0.99)	(0.96; 0.98)	(0.86; 0.92)
*a*_1_	1 ± 0.09	1 ± 0.08	1 ± 0.13
	(0.96; 1.04)	(0.96; 1.04)	(0.94; 1.06)
*a*_0_	0 ± 7.48	0 ± 7.51	0 ± 4.12
	(−3.79; 3.79)	(−3.80; 3.80)	(−2.09; 2.09)
Mean ± SD (*P* of *t*-test) of subjects with impairment			
*R* ^2^	0.90 ± 0.07	0.75 ± 0.20	0.40 ± 0.24
	(*P* < 0.001)	(*P* < 0.001)	(*P* < 0.001)
*a*_1_	0.77 ± 0.18	0.70 ± 0.29	0.42 ± 0.24
	(*P* < 0.001)	(*P* < 0.001)	(*P* < 0.001)
*a*_0_	6.72 ± 11.21	0.17 ± 9.69	−0.09 ± 6.40
	(*P* = 0.040)	(*P* = 0.953)	(*P* = 0.961)

**Table 3:** Analysis of reliability: results of intraclass correlation coefficients.

ICC	Hip	Knee	Ankle
Subjects with impairment			
*R* ^2^	0.80	0.84	0.84
*a*_1_	0.92	0.95	0.88
*a*_0_	0.99	0.97	0.97
